# A Phase 3, Randomized, Placebo-controlled Trial of Filgrastim in Patients with Haematological Malignancies Undergoing Matched-related Allogeneic Bone Marrow Transplantation

**DOI:** 10.1111/j.1753-5174.2008.00013.x

**Published:** 2008-12

**Authors:** Peter Ernst, Andrea Bacigalupo, Olle Ringdén, Tapani Ruutu, Hans J Kolb, Susan Lawrinson, Tomas Skacel

**Affiliations:** * Division of Haematology, Haukeland University HospitalBergen, Norway; † Ematologia—Centro Trapianti di Midollo OsseoGenoa, Italy; ‡ Huddinge HospitalHuddinge, Sweden; § Department of Medicine, Helsinki University Central HospitalHelsinki, Finland; ¶ Klinikum GrosshadernMunich, Germany; ** Department of Biostatistics, Amgen LtdUxbridge, UK; †† Medical Affairs, Amgen (Europe) GmbHZug, Switzerland; ‡‡ Department of Internal Medicine—Hemato-onkology, University HospitalBrno, Czech Republic

**Keywords:** Allogeneic Bone Marrow Transplant, Filgrastim, Neutrophils, Graft-versus Host Disease, Randomized Clinical Trial, Survival

## Abstract

**Introduction:**

Recombinant granulocyte colony-stimulating factor (G-CSF) may aid engraftment post high-dose chemo-/radiotherapy in patients with haematological malignancies undergoing allogeneic bone marrow transplantation (BMT); however, the effects of G-CSF on graft-versus-host disease (GvHD), relapse, and survival are not well defined.

**Methods:**

In this double-blind, randomized, placebo-controlled, multicentre, phase 3 study, the effects of the G-CSF Filgrastim on neutrophil and platelet recovery, and on clinical outcomes were evaluated. Patients (12–55 years) receiving an allogeneic BMT for a haematological malignancy were randomized to receive Filgrastim 5 µg/kg or placebo. Study treatment was continued until patients achieved an absolute neutrophil count (ANC) ≥0.5 × 10^9^/L, or until day 42.

**Results:**

Fifty-one patients (Filgrastim, N = 25; placebo, N = 26) were evaluable. Patients treated with Filgrastim had significantly faster engraftment with ANC ≥0.5 × 10^9^/L being achieved after a median (range) of 15.0 (1.0–22.0) days vs. 19.0 (15.0–28.0) days for placebo (*P*< 0.0001). The incidence of GvHD was comparable for both groups. During the limited follow-up (2 years), Filgrastim had no adverse effect on mortality and possibly reduced the rate of relapse.

## Introduction

The success of allogeneic haemopoietic stem cell transplantation is influenced by several factors such as: efficiency of the procedure to eradicate disease, age of the patient, immune reactions following transplantation of cells, and adverse reactions to conditioning treatment. In patients with chronic myeloid leukaemia, it has been shown that mortality post-bone marrow transplant (BMT) can vary from 22% to 72% at 5 years depending on the number of adverse risk factors [1]. Additional risk factors for mortality include infections that arise as a result of neutropenia and prolonged immune deficiency. Recombinant human granulocyte colony-stimulating factor (G-CSF) or granulocyte macrophage colony-stimulating factor (GM-CSF) may be administered after allogeneic transplantation of either bone marrow or peripheral blood stem cells to enhance engraftment [2–7]. Some authors report that patients receiving growth factors post allogeneic stem cell transplant experience faster neutrophil recovery and no adverse effects in terms of graft versus host disease (GvHD) [4, 8]. Nevertheless, the effects of G-CSF on longer-term outcomes in this setting are not well defined and have been the subject of debate [9–11]. Two retrospective analyses suggest that G-CSF treatment after allogeneic stem cell transplant may have a negative impact on outcome with increased risk of GvHD and reduced survival [10, 12].

With the aim of providing further information on the use and safety of G-CSF in this setting, we have returned to a previously unpublished phase 3, placebo-controlled trial of Filgrastim administered after allogeneic BMT in patients with haematological malignancies. Although the study was terminated in 1998 due to slow recruitment, the prospective nature of the data collected means that the present paper contributes to our knowledge about the safety of G-CSF in this setting.

## Patients and Methods

### Patients

This trial was conducted in compliance with the Declaration of Helsinki. The ethical committee at each participating site approved the study protocol. Written informed consent was obtained prior to study entry.

Eligible patients were aged 12–55 years with a diagnosis of acute lymphoblastic leukaemia (ALL) or lymphoblastic lymphoma (defined as T convoluted [Lukes-Collins Classification], lymphoblastic lymphoma [Kiel, Rappaport Classification], or undifferentiated [Lukes-Collins Classification]) who were in complete remission; high-risk non-Hodgkin's lymphoma (NHL) (Kiel, Rappaport Classification) in second remission; acute myeloid leukaemia (AML) in first or second remission; or chronic myeloid leukaemia (CML) in a chronic or accelerated phase. Patients had to have an Eastern Co-operative Oncology Group (ECOG) score of 0–2 or a Karnofsky score from 100–60.

Patients were scheduled to receive at least 2 × 10^8^ nuclear cells/kg from an HLA-matched sibling bone marrow donor following either: cyclophosphamide 60 mg/kg/day for 2 days (total dose 120 mg/kg) and total body irradiation (at least 1,000 cGy); or etoposide 60 mg/kg for 1 day (total dose 60 mg/kg) and total body irradiation 1,200 cGy; or busulphan 4 mg/kg for 4 days (total dose 16 mg/kg) and cyclophosphamide at 60 mg/kg for 2 days (total dose 120 mg/kg) with or without etoposide 30–45 mg/kg for 1 day (total dose 30–45 mg/kg). Patients were required to have serum creatinine and serum bilirubin levels less than 2.5 times the upper limit of normal.

Patients with a history of another malignancy, other than adequately treated carcinoma of the skin or cervical cancer (stage I), were ineligible, as were patients with active infections, and patients who had used antimicrobials within 72 hours prior to treatment. Congestive heart failure (NYHA class III–IV) precluded entry to the study, as did uncontrolled hypertension, multifocal cardiac arrhythmias or unstable angina. Pregnant or lactating women were also excluded, and those of childbearing age were required to have been practicing adequate contraception.

### Study Drug

Filgrastim and an identically packaged placebo for subcutaneous administration were manufactured and packaged by Amgen Inc. (Thousand Oaks, CA, USA) and distributed through F. Hoffmann-LaRoche (Basel, Switzerland). The Filgrastim preparation was a sterile aqueous buffered protein solution containing 0.3 mg/mL of Filgrastim. The placebo preparation contained the same sterile aqueous buffered protein solution without Filgrastim.

### Study Design

This was a phase 3, multicentre, randomized, double-blind, placebo-controlled study. Randomization was stratified by age (<18 years and ≥18 years) and remission status (first complete remission and subsequent remissions) at each centre. Patients were randomly assigned using Pocock and Simon's minimization procedure at a 1:1 ratio to receive bolus injections of either Filgrastim at 5 µg/kg/day or placebo for up to 42 days beginning on the day of allogeneic BMT.

Infection prophylaxis, parenteral feeding and other supportive care were performed according to the policies of the individual study site. Neutropenic patients were treated with a protocol defined regimen or according to local practice. Patients with engraftment failure on day 35 could be unblinded and treated with open-label Filgrastim for engraftment failure (these patients were censored on day 35 for the primary endpoint). Engraftment failure was defined as an absence of absolute neutrophil count (ANC) ≥0.5 × 10^9^/L on day 35 for 3 consecutive days and an absence of reduced transfusion requirements for platelets and red blood cells. Study medication was to be discontinued if the ANC was ≥1.0 × 10^9^/L for 3 consecutive days and reintroduced when ANC subsequently dropped below 1.0 × 10^9^/L for 2 consecutive days. All patients received GvHD prophylaxis as per standard protocol at each centre (cyclosporine plus methotrexate in all cases). If no GvHD was present, this regimen was tapered off at day 100. GvHD was treated using the standard individual centre regimen.

### Efficacy Endpoints and Safety Analysis

The predetermined primary efficacy endpoint was the time to ANC of ≥0.5 × 10^9^/L, and the time to ANC ≥1.0 × 10^9^/L was also evaluated. The secondary efficacy endpoints for the immediate post-transplant period were: time to discharge from semi-sterile conditions; the number of days of fever, neutropenic fever, and antibiotic use (excluding prophylactic antibiotics); the duration of neutropenia; the time to platelet recovery of ≥25 × 10^9^/L and ≥50 × 10^9^/L, and the number of days with platelets <25 × 10^9^/L and <50 × 10^9^/L.

After hospital discharge, patients were followed up at weekly intervals for 6 weeks, alternate weeks thereafter for 1 month, and every 3 months for 2 years following allogeneic BMT. During this time period, data were collected on GvHD, and safety was assessed as the incidence and severity of adverse events and as changes in laboratory tests. Additional parameters included overall survival (defined as the time from transplantation day until death or until the day of last follow-up) and relapse-free survival (defined as the time from transplantation day until clinical diagnosis of relapse). Conventional haematological criteria were used to define remission status. The criteria for remission status included establishment of a normal bone marrow cell karyotype, and immunoglobulin gene or T-cell receptor gene rearrangement.

### Statistical Analysis

At least 40 evaluable patients (20 per treatment group) were required to ensure a probability of at least 80% of detecting a difference of at least 3.6 days between treatment groups in the primary efficacy endpoint of time to ANC recovery (ANC ≥ 0.5 × 10^9^/L) using the log-rank test with a two-tailed significance level of 5% and assuming a standard deviation of 3.8 days. The target of 100 evaluable patients (50 per treatment group) was set to provide 80% probability of detecting a difference of 3.6 days between groups in the secondary endpoint of time to release from semi-sterile conditions, using the log-rank test with a two-tailed significance level of 5% and assuming a standard deviation of 5.8 days.

An interim analysis was planned for the point when 40 patients had been followed for at least 100 days post BMT. In this analysis, the time to ANC recovery was compared between groups using the log-rank test and a two-tailed significance level of 4.8%.

Quantitative parameters were summarized by the mean, standard deviation, median, upper and lower quartiles, and the range (minimum and maximum values). Non-parametric statistical analysis methods were used to compare treatment groups. For all endpoints, excluding time-to-event endpoints, the treatment groups were compared using the Mann-Whitney test. For time-to-event endpoints, the data were analysed using the log-rank test.

## Results

### Interim Analysis

The study was initiated in January 1993 and an interim analysis was performed as planned in March 1996. This analysis showed a statistically significant and clinically relevant difference in favour of Filgrastim over placebo in the time to ANC recovery. The median (range) time to ANC ≥ 0.5 × 10^9^/L was 15.0 (1.0–22.0) days for Filgrastim (N = 18) compared with 19.0 (15.0–28.0) days for placebo (N = 19) (*P* < 0.0001). No statistically significant difference was observed in the median time to discharge from semi-sterile conditions (Filgrastim: 24.0 [14.0–43.0] vs. placebo: 28.0 [19.0 vs. 43.0] days; *P* = 0.8213).

### Final Analysis—Patient Disposition and Baseline Characteristics

Between January 1993 and November 1996, a total of 66 patients were enrolled in the study from seven centres located in Germany (two centres), Saudi Arabia (two centres), Finland, Italy, and Sweden. Recruitment was slow and no further patients were randomized after November 1996. Patients were followed up until March 1998, when the study was closed.

Fifteen patients had major protocol violations: Filgrastim treatment not according to protocol (N = 5); nuclear cells <2.0 × 10^8^/L (N = 3); missing data (N = 2); two bone marrow transfusions (N = 1); no BMT (N = 1); inappropriate conditioning regimen (N = 1); inappropriate discharge conditions (N = 1); received commercial drug (N = 1). These patients were excluded from the efficacy and safety analyses, which consequently contained 51 patients (Filgrastim, N = 25; placebo, N = 26).

Patient baseline demographics and disease characteristics are shown in Table 1. The treatment groups were generally well-balanced, although the placebo group had a higher proportion of men and higher mean weight. The most common diagnosis in both groups was ALL, and the most common remission status was first complete remission. The stratification of patients by age and remission status resulted in a good balance between the groups with regard to these parameters.

**Table 1:** Baseline demographics and disease characteristics

	Filgrastim (N = 25)	Placebo (N = 26)
Men, N (%)	13 (52%)	21 (81%)
Mean (SD) age (years)	28.2 ± 10	27.4 ± 10
Mean (SD) weight (kg)	59.8 ± 15	68.7 ± 16
Diagnosis, N (%)		
Acute lymphatic leukaemia (ALL)	18 (72%)	17 (65%)
Biphenotypic leukaemia	0 (0)	1 (4%)
Acute myeloid leukaemia (AML)	2 (8%)	3 (12%)
Chronic myeloid leukaemia (CML)	5 (20%)	4 (15%)
Lymphoblastic lymphoma	0 (0)	1 (4%)
Remission Status, N (%)		
Complete remission*	5 (20%)	8 (31%)
First complete remission	13 (52%)	11 (42%)
Second complete remission	2 (8%)	2 (8%)
Third complete remission	0 (0)	1 (4%)
First chronic phase	2 (8%)	1 (4%)
Accelerated phase	1 (4%)	1 (4%)
Second chronic phase	1 (4%)	0 (0)
None provided	1 (4%)	2 (8%)

* Number of complete remission not specified for these patients.

### Neutrophil Recovery and Related Parameters

**Figure 1:**
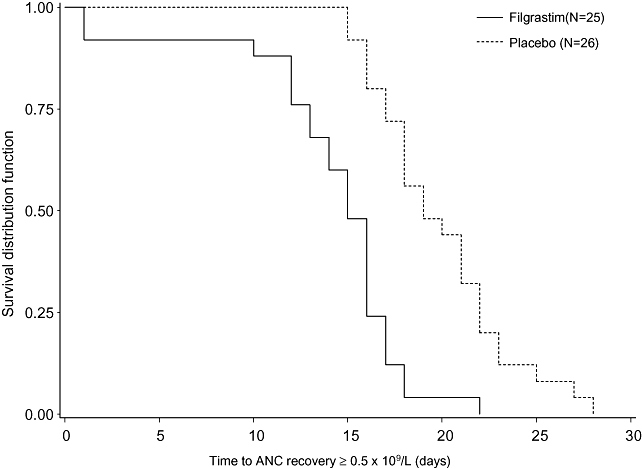
Kaplan-Meier analysis of time to recovery of absolute neutrophil count in patients receiving filgrastim vs. placebo.

In the final analysis of the primary endpoint, the median (Q1, Q3) number of days to achieve ANC ≥ 0.5 × 10^9^/L was 15.0 (13.0, 16.0) days in the Filgrastim group, compared to 19.0 (17.0, 22.0) days in the placebo group. Faster recovery of neutrophil counts in patients receiving Filgrastim was also evident in a time to event analysis (Figure 1). Similarly, the median (Q1, Q3) number of days to achieve ANC ≥ 1.0 × 10^9^/L was 16.0 (15.0, 17.0) days in the Filgrastim group, compared to 22.0 (21.0, 28.0) days in the placebo group. For both variables, the differences between Filgrastim and placebo were statistically significant (*P* < 0.0001 vs. placebo; Table 2).

**Table 2:** Neutrophil recovery and related parameters

	Filgrastim (N = 25)		Placebo (N = 26)
Time to recovery of ANC ≥ 0.5 × 10^9^/L	(N = 25)		(N = 25)
Median (range), days	15.0 (1.0–22.0)		19.0 (15.0–28.0)
Q1, Q3	13.0, 16.0		17.0, 22.0
Difference		4 days	
*P* value		*P* < 0.0001	
Time to recovery of ANC ≥ 1.0 × 10^9^/L	(N = 25)		(N = 25)
Median (range), days	16.0 (1.0–23.0)		22.0 (15.0–37.0)
Q1, Q3	15.0, 17.0		21.0, 28.0
Difference		6 days	
*P* value		*P* < 0.0001	
Time to discharge from semi-sterile conditions	(N = 25)		(N = 26)
Median (range), days	25.0 (14.0–43.0)		28.0 (10.0–43.0)
Q1, Q3	22.0, 30.0		25.0, 33.0
Difference		3 days	
*P* value		*P* = 0.3304	
Days with neutropenic fever	(N = 25)		(N = 26)
Median (range)	5.0 (0.0–16.0)		6.0 (0.0–18.0)
Q1, Q3	1.0, 16.0		0.0, 18.0
Difference		1 day	
*P* value		*P* = 0.2214	
Days on antibiotics	(N = 24)		(N = 26)
Median (range)	11.5 (0.0–42.0)		18.0 (0.0–43.0)
Q1, Q3	0.0, 22.5		0.0, 28.0
Difference		6.5 days	
*P* value		*P* = 0.1791	

The median time to discharge from semi-sterile conditions, median number of days with neutropenic fever and median number of days of IV antibiotic use, were all numerically lower with Filgrastim than with placebo. The differences between treatment groups did not, however, reach statistical significance for any of these endpoints (Table 2).

### Platelet Recovery

It transpired that investigators had stopped recording platelet counts after ANC recovery—an event that occurred more rapidly for those receiving Filgrastim. Consequently, many patients with “low” platelet counts at last assessment may in fact have reached recovery thresholds within the 42-day follow-up period, but this is impossible to determine, thus the platelet data are confounded and are not reported in this manuscript.

### Graft-versus-host Disease

About half of the patients in both treatment arms remained free of acute GvHD throughout the whole study (Table 3). Among those patients that exhibited acute GvHD, most experienced maximum grade 1 disease but a few patients in each group displayed grade 2 or 3 disease (Table 3). The number of days to first presentation with acute GvHD and the number of days to worst acute GvHD were also similar between the treatment groups. Overall, the data indicate no difference in the incidence and severity of acute GvHD between patients receiving Filgrastim and those receiving placebo. The incidence of physician-reported chronic GvHD was similar between the groups (Table 3).

**Table 3:** Graft versus host disease (GvHD)

Treatment group	Filgrastim (N = 25)	Placebo (N = 26)
Patients with acute GvHD, N (%)	13 (52%)	12 (46%)
Number of days to first acute GvHD		
Median (range)	15.0 (7.0–47.0)	18.5 (5.0–59.0)
Q1, Q3	(14.0, 22.0)	(15.0, 31.0)
Worst acute GvHD, N (%)*		
N	25	25
Grade 1	10 (40%)	7 (28%)
Grade 2	2 (8%)	4 (16%)
Grade 3	1 (4%)	1 (4%)
Grade 4	0 (0)	0 (0)
No GvHD	12 (48%)	13 (52%)
Number of days to worst acute GvHD		
Median (range)	21.0 (7.0–47.0)	19.0 (5.0–59.0)
Q1, Q3	14.0, 29.0	15.0, 36.0
Patients with chronic GVHD,† N (%)	10 (40%)	12 (46%)

* Acute GvHD was graded 1–4 depending on degree of organ involvement. Grade 1 signified no gut or liver involvement and no decrease in clinical performance in the presence of macularpapular skin rash covering up to 50% of body surface, and grade 4 denoted significant gut and liver involvement, an extreme decrease in clinical performance and desquamation of the skin.

† Physician-reported.

### Overall Survival and Time to Relapse

During the observation period (maximum of 2 years post-BMT), 4 (16%) patients in the Filgrastim group and 8 (31%) patients in the placebo group died (Table 4). All deaths occurred within 1 year of BMT. No deaths were related to Filgrastim treatment. Only 1 (4%) patient in the Filgrastim group had a relapse, compared to 8 (31%) patients in the placebo group. The patient in the Filgrastim group had ALL at baseline, as did five of the patients who relapsed in the placebo group. Other baseline diagnoses in this group were AML (N = 2) and biphenotypic leukaemia (N = 1).

**Table 4:** Deaths and causes of death according to treatment group

	Treatment group	
	
	Filgrastim (N = 25)	Placebo (N = 26)
Patients with ≥1 cause of death, N (%)	4 (16%)	8 (31%)
Causes of death, N		
Heart failure	1	
Haemorrhage	1	
Multiorgan failure	1	
Sepsis	1	
Disease progression		3
Leukaemia		1
Neurological disorders		1
Respiratory tract infection		1
Unknown diagnosis/not specified	1	2

NB. One patient in the Filgrastim group had two designated causes of death—heart failure and sepsis.

### Safety and Tolerability

Adverse events occurred in similar proportions of the Filgrastim and placebo groups: (19/25 [76%] with Filgrastim vs. 19/26 [73%] with placebo). The only event occurring with 5% greater frequency in the Filgrastim group than the placebo group was fever (6/25 [24%] vs. 4/26 [15%], respectively). Three adverse events in the Filgrastim group (weight change, liver disease, and abnormal renal function) and six events in the placebo group (fever [N = 2], weight change, nausea, vomiting, and musculoskeletal pain) were reported as possibly related to treatment; none of these events were severe or life threatening. The incidence of life-threatening adverse events was 16% (4/25) in the Filgrastim group vs. 8% (2/26) for placebo, none were considered related to study treatment.

## Discussion

In this randomized study, patients receiving Filgrastim post BMT had faster ANC recovery than those receiving placebo. Furthermore, the study suggests no difference in time to discharge from semi-sterile conditions. ANC is the main driver of discharge from semi-sterile conditions, but we realize that other factors such as oral nutrition and ability to take drugs by mouth may influence this decision. Adverse events occurred at a similar rate in the Filgrastim and placebo groups.

The present data broadly reflect previous findings with regard to the effects of growth factors on ANC recovery post allogeneic BMT. For example, in a trial of 40 patients randomized to 8 µg/kg/day GM-CSF (N = 20) or placebo (N = 20), Singhal et al. reported a significantly faster neutrophil recovery [8]. Furthermore, in a randomized double-blind trial of G-CSF 10 µg/kg/day (N = 26) vs. placebo (N = 24) administered after allogeneic BMT, G-CSF also resulted in significantly more rapid neutrophil recovery (11 vs. 15 days for placebo, *P* = 0.008) [4]. More recently, a retrospective database analysis by Khoury et al. showed a statistically significant reduction in time to neutrophil engraftment for patients receiving G-CSF following transplantation of bone marrow from unrelated donors compared to no growth factor support (median 16 vs. 20 days; *P* < 0.001) [7].

Our main reason for returning to this prospective study was to examine data on GvHD and survival. Two retrospective analyses have questioned whether G-CSF may be associated with a higher incidence of immunologic reactions [10, 12]. For example, the retrospective European analysis by Ringden et al., identified a possibly increased incidence of GvHD and transplant-related mortality in patients (N = 1,789) receiving G-CSF soon after allogeneic BMT, as well as reduced survival and leukaemia-free survival [10]; however no increased rate of GvHD was observed for patients receiving G-CSF following peripheral blood stem cell transplant. In the previously mentioned randomized study by Singhal, GM-CSF was associated with no deleterious effect on acute or chronic GvHD, relapse or survival after 5-year follow-up [8], while the study by Bishop also showed no difference between G-CSF and placebo in acute GvHD [4]. Furthermore, a meta-analysis of 18 studies involving 1,198 patients showed that G-CSF had no effect on the incidence of acute GvHD, chronic GvHD, transplant-related mortality or survival [13]. These findings have since been corroborated by another retrospective database analysis including 1,435 bone marrow recipients [7] and a further meta-analysis of studies in patients undergoing either allogeneic or autologous stem cell transplant [14].

Within the limits of the current dataset there was no difference in the incidence or severity of acute GvHD for patients receiving Filgrastim compared with placebo, nor was there any adverse effect on mortality or relapse. Fewer patients experienced relapse in the Filgrastim group compared with placebo. Any potential benefit from Filgrastim in terms of relapse could be the result of stem cell competition: the earlier engraftment and graft function experienced with Filgrastim support may favour donor cells competing with leukaemia cells of the recipient [15]. It is also a possibility, however, that the placebo group contained a greater proportion of patients at high risk of relapse. For example, this arm was more heavily weighted with males, who tend to have poorer outcomes than females in allogeneic BMT. The effect of Filgrastim on relapse needs to be explored in a study adequately powered for this endpoint.

Our findings are limited by early termination of the study and the small number of patients randomized. The slow recruitment that forced termination may have been related to an unwillingness to randomize patients to placebo; widespread use of Filgrastim in this setting could have meant that it was already accepted as the standard of care. Furthermore, mortality and relapse data must be interpreted within the context of the high proportion of censored patients and the limited follow-up period. Although clinical practice may have changed since this study was conducted, these prospective, blinded data are not subject to the potential biases of retrospective studies, and provide further information on the safety of G-CSF post allogeneic BMT.

In conclusion, the present study provides prospective data indicating that the G-CSF Filgrastim is associated with significantly more rapid neutrophil recovery than placebo in patients undergoing allogeneic BMT. Filgrastim had no detrimental effect on acute GvHD, while the potential effect of Filgrastim therapy with regard to relapse requires further investigation.
